# First Identification of 5,11-Dideoxytetrodotoxin in Marine Animals, and Characterization of Major Fragment Ions of Tetrodotoxin and Its Analogs by High Resolution ESI-MS/MS

**DOI:** 10.3390/md11082799

**Published:** 2013-08-06

**Authors:** Mari Yotsu-Yamashita, Yuka Abe, Yuta Kudo, Raphael Ritson-Williams, Valerie J. Paul, Keiichi Konoki, Yuko Cho, Masaatsu Adachi, Takuya Imazu, Toshio Nishikawa, Minoru Isobe

**Affiliations:** 1 Graduate School of Agricultural Science, Tohoku University, Sendai 981-8555, Japan; E-Mails: y.abe.woodstock@gmail.com (Y.A.); b3ad1305@s.tohoku.ac.jp (Y.K.); konoki@m.tohoku.ac.jp (K.K.); choyuko@m.tohoku.ac.jp (Y.C.); 2 Department of Biology, University of Hawaii at Manoa, 2540 Campus Road, Dean Hall, Honolulu, HI 96822, USA; E-Mail: rrw33@hawaii.edu; 3 Smithsonian Marine Station at Fort Pierce, 701 Seaway Drive, Fort Pierce, FL 34949, USA; E-Mail: Paul@si.edu; 4 Graduate School of Bioagricultural Sciences, Nagoya University, Chikusa, Nagoya 464-8601, Japan; E-Mails: madachi@agr.nagoya-u.ac.jp (M.A.); imazu.takuya@b.mbox.nagoya-u.ac.jp (T.I.); nisikawa@agr.nagoya-u.ac.jp (T.N.); 5 Department of Chemistry, National Tsing Hua University, 101, Section 2, Kuang-Fu Road, Hsinchu 30013, Taiwan; E-Mail: minoru@mx.nthu.edu.tw

**Keywords:** tetrodotoxin, LC-MS/MS, 5,11-dideoxytetrodotoxin, biosynthesis

## Abstract

Even though tetrodotoxin (TTX) is a widespread toxin in marine and terrestrial organisms, very little is known about the biosynthetic pathway used to produce it. By describing chemical structures of natural analogs of TTX, we can start to identify some of the precursors that might be important for TTX biosynthesis. In the present study, an analog of TTX, 5,11-dideoxyTTX, was identified for the first time in natural sources, the ovary of the pufferfish and the pharynx of a flatworm (planocerid sp. 1), by comparison with totally synthesized (−)-5,11-dideoxyTTX, using high resolution ESI-LC-MS. Based on the presence of 5,11-dideoxyTTX together with a series of known deoxy analogs, 5,6,11-trideoxyTTX, 6,11-dideoxyTTX, 11-deoxyTTX, and 5-deoxyTTX, in these animals, we predicted two routes of stepwise oxidation pathways in the late stages of biosynthesis of TTX. Furthermore, high resolution masses of the major fragment ions of TTX, 6,11-dideoxyTTX, and 5,6,11-trideoxyTTX were also measured, and their molecular formulas and structures were predicted to compare them with each other. Although both TTX and 5,6,11-trideoxyTTX give major fragment ions that are very close, *m/z* 162.0660 and 162.1020, respectively, they are distinguishable and predicted to be different molecular formulas. These data will be useful for identification of TTXs using high resolution LC-MS/MS.

## 1. Introduction

Tetrodotoxin (TTX) [1,2,3] is a well-known voltage-gated Na^+^ channel blocker [4,5], found in a wide variety of marine animals, such as pufferfish [6], crabs [7,8], flatworms [9,10], snails [11], starfish [12], blue-ringed octopus [13], and sea slugs [14], and also found in some species of amphibians, frogs [15] and newts [16]. TTX in marine animals is thought to come from external origin, since TTX-producing marine bacteria have been reported [17,18], and pufferfish has been experimentally shown to have an ability to accumulate TTX administrated dietarily [19,20] and intramuscularly [21,22,23]. However, the origin of TTX in amphibians is still controversial [24], and the biosynthetic pathways of TTX in both marine and terrestrial organisms still remain to be clarified. 

**Figure 1:**
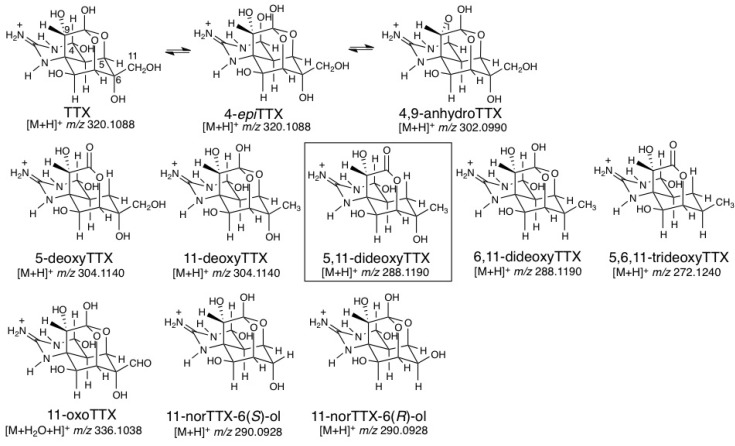
The structures of natural tetrodotoxin (TTX) analogs and the calculated masses for their [M + H]^+^ or [M + H_2_O + H]^+^.

We have isolated and determined the structures of many natural TTX analogs found in pufferfish [25,26] and amphibians (Figure 1). TTX analogs found in pufferfish can be classified into four groups: (1) Analogs chemically equivalent to TTX (4-*epi*TTX and 4,9-anhydroTTX) [27], (2) Deoxy analogs (5-deoxyTTX [28], 11-deoxyTTX [29,30], 6,11-dideoxyTTX [31], and 5,6,11-trideoxyTTX [32]), (3) 11-CH_2_OH oxidized analog (11-oxoTTX) [33], and (4) C11 lacking analogs (11-norTTX-6(*S*)-ol [34] and 11-norTTX-6(*R*)-ol [35]). Amphibian specific analogs have also been found, such as chiriquitoxin [36,37] in the central American toads, *Atelopus chiriquiensis*, *A. limosus* and *A. glyphus*, and 1-*N-*hydroxy-5,11-dideoxyTTX [38] in the North American newt, *Taricha granulosa*, found by Kotaki and Shimizu, and 6-*epi*TTX [30], 8-*epi*-5,6,11-trideoxyTTX and 1-*N*-hydroxy-8-*epi*-5,6,11-trideoxytTTX [39] in the Japanese newt, *Cynops ensicauda popei*. These natural analogs are useful for predicting the biosynthetic pathway of TTX.

Recently, LC-MS has been a common analytical method for TTX [40]. We have developed a LC-MS method to detect almost all of the major natural analogs of TTX using HILIC (hydrophilic interaction chromatography) mode for separation for the first time in 2006 [41]. We also reported that TTX and its analogs commonly give the major fragment ion at *m/z* 162 using ESI-triple quadrupole mass spectrometer [42]. This fragment ion has been widely used to detect TTX and its analogs in MRM (multiple reaction monitoring) mode by detecting *m/z* 320/162 transition in tandem mass spectrometry [40,43,44]. However, the structures of the major fragment ions of TTX and its analogs, including this *m/z* 162 ion, have not been determined using a high resolution mass spectrometer. 

Here we report identification of a new deoxy analog of TTX, 5,11-dideoxyTTX, in two marine animals by comparison of retention times and fragmentation patterns with those of totally synthesized (−)-5,11-dideoxyTTX using high resolution ESI-LC-MS/MS. Nishikawa and Isobe *et al.*, reported stereocontrolled synthesis of (−)-5,11-dideoxyTTX as an unnatural analog of TTX in 1999 [45,46,47], and Isobe *et al.* developed an analytical method for TTXs using LC-quadrupole-time of flight (Q-TOF) MS [48]. Furthermore, Adachi and Nishikawa *et al.* recently reported an improved synthesis of this compound [49]. Although Kotaki and Shimizu [38] isolated 1-*N*-hydroxy-5,11-dideoxyTTX from the newt, *Taricha granulosa*, 5,11-dideoxyTTX has not been previously found in either marine or terrestrial animals. Based on identification of a series of deoxy analogs including 5,11-dideoxyTTX, we predict stepwise oxidation in the later stages of biosynthesis of TTX. Furthermore, we also characterized major fragment ions of TTX, 5,11-dideoxyTTX, 6,11-dideoxyTTX, and 5,6,11-trideoxyTTX using high resolution ESI-MS spectrometer to compare with each other. These data are useful for identification of TTX and its analogs using high resolution ESI-LC-MS/MS.

## 2. Results

### 2.1. LC-MS Chromatographic Properties of Authentic 5,11-DideoxyTTX and 6,11-DideoxyTTX, and Characterization of Their Major Fragment Ions

**Figure 2:**
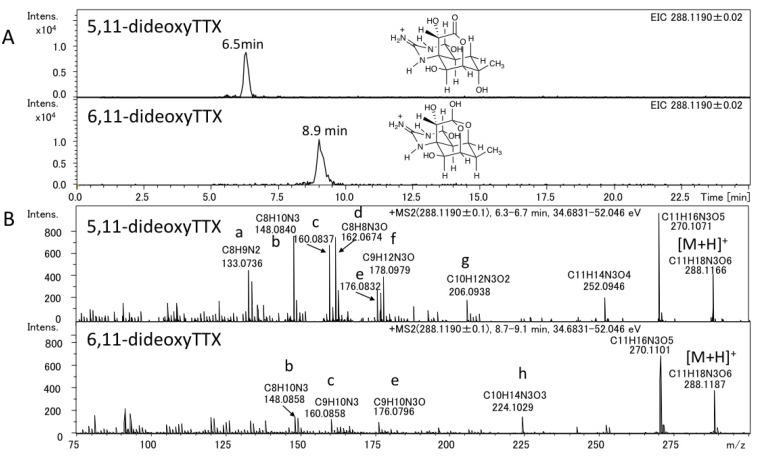
LC-MS chromatograms of the mixture of the authentic (−)-5,11-dideoxyTTX (2.0 ng) and 6,11-dideoxyTTX (3.7 ng) (**A**) and their fragment ion spectra (**B**). LC was performed using 16 mM ammonium formate in water/acetonitrile/formic acid (30:70:0.002, v/v) as a mobile phase at the flow rate of 0.2 mL/min at 25 °C, with a 2.0 i.d. × 150 mm (5 µm) TSK-GEL Amide-80 column.

**Figure 3:**
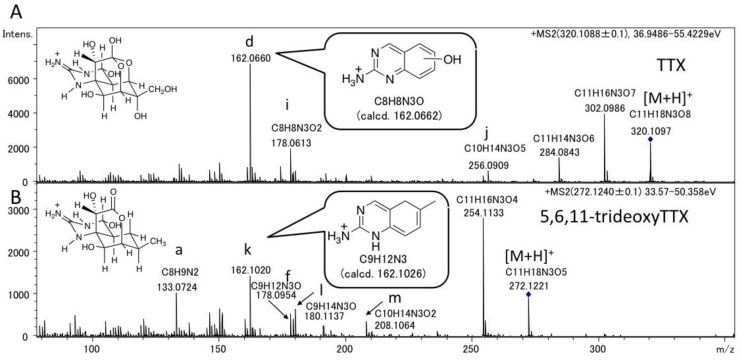
The fragment ion spectra of authentic TTX (**A**) (2.0 ng) and 5,6,11-trideoxyTTX (**B**) (0.70 ng) with the predicted structures of the fragment ions at *m/z* 162. (−OH) in the structure of the fragment ion (d) indicates the presence of a hydroxyl group anywhere in the ring. The structures of other fragment ions with alphabets are shown in Figure 4.

**Figure 4:**
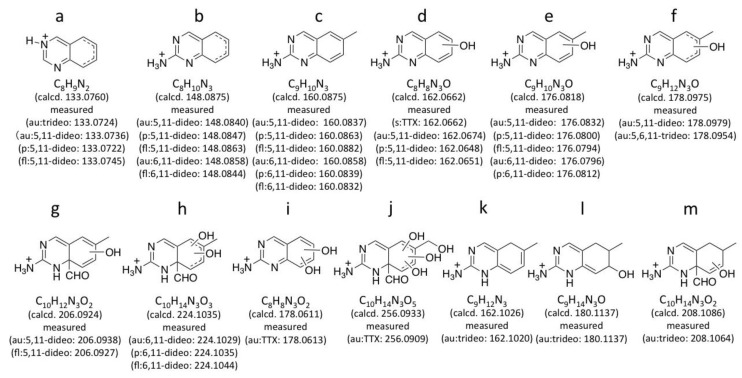
The representative possible structures of the major fragment ions of 5,11-dideoxyTTX (5,11-dideo), 6,11-dideoxyTTX (6,11-dideo), TTX, and 5,6,11-trideoxyTTX (5,6,11-trideo) and their measured and calculated masses shown in the fragment ion spectra in Figure 2B, Figure 3, Figure 5B, Figure 6B. The dotted line in the structure indicates presence of a double bond anywhere. (−OH) in the structure indicates presence of a hydroxyl group anywhere in the ring. “au”, “p”, and “fl” denote the ions shown in the fragment ion spectra of authentic (au) standards (Figure 2B, Figure 3), pufferfish (p) (Figure 5B), and flatworm (fl) (Figure 6B) samples, respectively. These structures were predicted considering the stability of conjugated double bonds.

The authentic (−)-5,11-dideoxyTTX (2.0 ng) (synthesized) [49] and 6,11-dideoxyTTX (3.7 ng) (purified from pufferfish [31]) were independently applied to high resolution ESI-LC-MS (Q-TOF MS) in AutoMS/MS mode. AutoMS/MS function automatically collects fragment spectra across each LC/MS peak. In this mode, both the Q1 scan mass chromatograms and the fragment ion spectra from the specific precursor ions ([M + H]^+^ *m/z* 288.1190 ± 0.1 for dideoxyTTX) are simultaneously obtained. In this experiment, TTXs were separated in HILIC mode as we reported previously [29,41,43,44]. The extract ion chromatograms (EICs) for [M + H]^+^ of these dideoxy analogs are shown in Figure 2A, their fragment ion spectra with the high resolution masses and predicted molecular formulas are shown in Figure 2B, and the fragment ion spectra of TTX and 5,6,11-trideoxyTTX are also shown in Figure 3 for comparison. The peaks of (−)-5,11-dideoxyTTX and 6,11-dideoxyTTX eluted separately at 6.5 min and 8.9 min, respectively (Figure 2A). In the fragment ion spectra of two dideoxyTTX analogs, dehydrated ions losing one and two waters were observed at *m/z* 270.1071 (calcd. 270.1084) and 252.0946 (calcd. 252.0978) for (−)-5,11-dideoxyTTX, and one water losing ion was observed at *m/z* 270.1101 (calcd. 270.1084) for 6,11-dideoxyTTX. The other major fragment ions for (−)-5,11-dideoxyTTX were detected at *m/z* 133.0736 (a), 148.0840 (b), 160.0837 (c), 162.0674 (d), 176.0832 (e), 178.0979 (f), and 206.0938 (g), and those for 6,11-dideoxyTTX were detected at *m/z* 148.0858 (b), 160.0858 (c), 176.0796 (e), and 224.1029 (h). The fragment ions (a), (d), (f), and (g) were specific to 5,11-dideoxyTTX, and (h) was specific to 6,11-dideoxyTTX, while (b), (c), and (e) were commonly shown for these two dideoxyTTX analogs (Figure 2B). In addition, the fragment ion (d) shown at *m/z* 162.0674 for (−)-5,11-dideoxyTTX was also shown for TTX at *m/z* 162.0660 (Figure 3A). The predicted structures based on the high resolution masses of these fragment ions for two dideoxyTTX are shown with those of TTX and 5,6,11-trideoxyTTX in Figure 4. The differences between measured and calculated masses for all the fragment ions (a)-(m) shown in Figure 2, Figure 3 were less than 3 mDa as shown in Figure 4.

### 2.2. Characterization of Major Fragment Ions of TTX and 5,6,11-TrideoxyTTX

For comparison with the dideoxyTTXs, the major fragment ions of TTX and 5,6,11-trideoxyTTX were also examined by LC-MS/MS in AutoMS/MS mode (Figure 3). TTXs mixture containing TTX (2.0 ng/injection) and 5,6,11-trideoxyTTX (0.70 ng/injection) prepared from the ovary of *Takifugu poecilonotus* was applied setting the precursor ions at *m/z* 320.1088 ± 0.1 for TTX, and *m/z* 272.1240 ± 0.1 for 5,6,11-trideoxyTTX. The major fragment ions from [M + H]^+^ ion of TTX (320.1088 ± 0.1) were detected at *m/z* 302.0986 ([M − H_2_O + H]^+^, calcd. 302.0983 for C_11_H_16_N_3_O_7_), 284.0843 ([M − 2H_2_O + H]^+^, calcd. 284.0877 for C_11_H_14_N_3_O_6_), 256.0909 (j), 178.0613 (i), and 162.0660 (d). The major fragment ions of 5,6,11-trideoxyTTX were detected at *m/z* 254.1133 ([M − H_2_O + H]^+^, calcd. 254.1135 for C_11_H_16_N_3_O_4_), 208.1064 (m), 180.1137 (l), 178.0954 (f), 162.1020 (k), and 133.0724 (a) (Figure 3). Interestingly, the fragment ion at *m/z* 162.0660 (d) (calcd. 162.0662 for C_8_H_8_N_3_O) of TTX was distinguished from that at 162.1020 (k) (calcd. 162.1026 for C_9_H_12_N_3_) of 5,6,11-trideoxyTTX. Similarly, the fragment ion at *m/z* 178.0613 (i) (calcd. 178.0611 for C_8_H_8_N_3_O_2_) of TTX was distinguished from that at *m/z* 178.0954 (f) (calcd. 178.0975 for C_9_H_12_N_3_O) of 5,6,11-trideoxyTTX. The possible structures for these fragment ions are shown in Figure 4. These structures were predicted considering the stability of conjugation of double bonds. The differences between measured and calculated masses were less than 4 mDa.

### 2.3. Identification and Quantitation of 5,11-DideoxyTTX in the Pufferfish, *Takifugu poecilonotus*, and Flatworm (Planocerid sp. 1)

**Figure 5:**
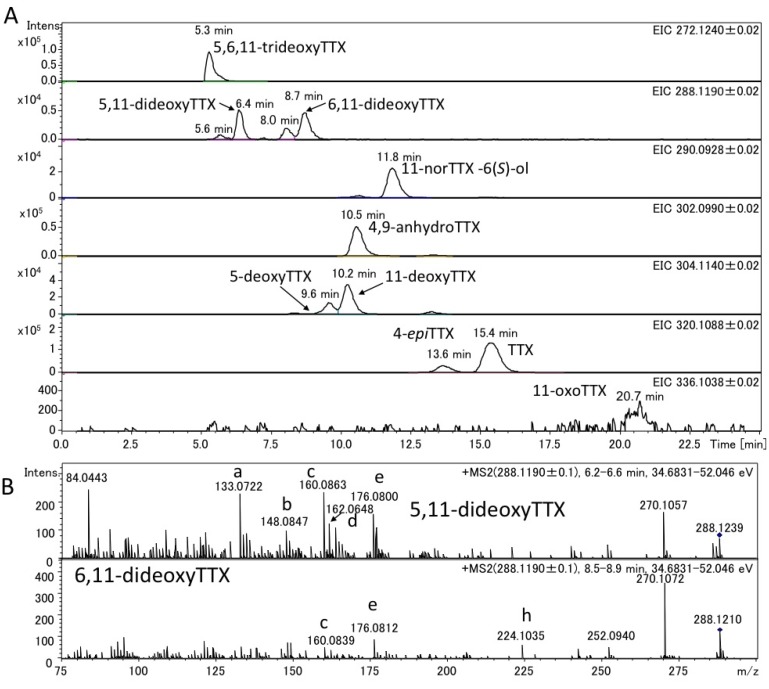
Extract ion chromatograms (EIC) of TTXs (**A**) and fragment ion spectra of 5,11-dideoxyTTX and 6,11-dideoxyTTX (**B**) in the semi-purified TTXs mixture fromthe pufferfish (*Takifugu poecilonotus*) ovary. The structures of the ions with alphabets are shown in Figure 4.

**Figure 6:**
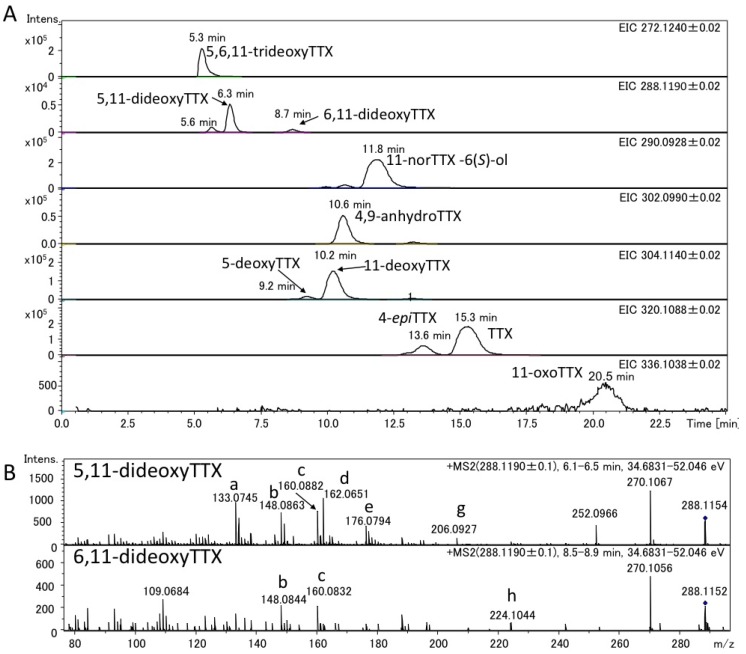
EIC of TTXs (**A**) and fragment ion spectra of 5,11-dideoxyTTX and 6,11-dideoxyTTX (**B**) in the semi-purified TTXs mixture from flatworm (planocerid sp. 1) pharynx. The structures of the ions with alphabets are shown in Figure 4.

The charcoal treated extracts from the pooled ovary of the Japanese pufferfish, *Takifugu poecilonotus*, and that of the pharynx of a flatworm (planocerid sp. 1) collected in Guam were applied to high resolution LC-MS. The applied sample solutions contained 4.0 ng and 7.0 ng of TTX in pufferfish and flatworm extracts, respectively. The EIC of 5,6,11-trideoxyTTX (272.1240 ± 0.02), dideoxyTTXs (288.1190 ± 0.02), 11-norTTX-6(*S*)-ol (290.0928 ± 0.02), 4,9-anhydroTTX (302.0990 ± 0.02), monodeoxyTTXs (304.1140 ± 0.02), TTX and 4-*epi*TTX (320.1088 ± 0.02), and 11-oxoTTX (336.1038 ± 0.02) in the extracts of the pufferfish and flatworm were shown in Figure 5A, Figure 6A, respectively. In these EIC, the peaks corresponding to 5,11-dideoxyTTX were shown at 6.4 min for pufferfish (Figure 5A) and at 6.3 min for flatworm (Figure 6A), almost agreeing with that of the authentic 5,11-dideoxyTTX (6.5 min) (Figure 3A). Probably, the smaller peaks at 5.6 min and 8.0 min in the EIC (288.1190 ± 0.02) are those of 4-*epi* types of 5,11-dideoxyTTX and 6,11-dideoxyTTX, respectively, since TTX analogs are usually present with 4-*epi* and 4,9-anhydro forms that are chemically in equilibrium to TTX form [1,2,3,27]. The applied amounts and contents of these TTX analogs in the samples were estimated based on the peak areas and shown in Table 1, since the good linearity of the relationship between dose and area size of TTX in this LC-Q-TOF MS system was confirmed at the range of 2–7000 pg (R = 0.9996). The flatworm contained 5,11-dideoxyTTX, approximately seven fold more than 6,11-dideoxyTTX, while the ovary of pufferfish, *T. poecilonotus*, contained these dideoxy analogs almost at the same level.

The fragment ion spectra obtained in AutoMS/MS mode setting the precursor ion at *m/z* 288.1190 ± 0.1 for dideoxyTTXs are shown for the pufferfish and flatworm in Figure 5B, Figure 6B, respectively. These fragment ion spectra were almost identical to those of authentic (–)-5,11-dideoxyTTX and 6,11-dideoxyTTX (Figure 3B), although some other ions were observed for the samples, probably due to the influence from impurity in the samples. For these fragment ions shown in Figure 5B, Figure 6B, the differences between measured and calculated masses were less than 4 mDa as shown in Figure 4. 

**Table 1:** The applied amount to LC-MS (Figure 5, Figure 6) and the contents of TTXs in *Takifugu poecilonotus* ovary and flatworm pharynx.

	*Takifugu poecilonotus* ovary			Flatworm (planocerid sp. 1) pharynx		
	Applied amount (Figure 5) (ng)	Contents (µg/g)	Ratio (mol/mol)	Applied amount (Figure 6) (ng)	Contents (µg/g)	Ratio (mol/mol)
TTX	4.0	130	100	7.0	1800	100
4- *epi*TTX	0.89	30	22	1.9	480	27
4,9-anhydroTTX	1.1	36	27	1.1	280	16
5-deoxyTTX	0.25	8.4	6.2	0.41	100	5.9
11-deoxyTTX	0.67	22	17	4.0	1000	57
6,11-dideoxyTTX	0.082	2.7	2.0	0.090	23	1.3
5,11-dideoxyTTX	0.065	2.2	1.6	0.66	170	9.4
5,6,11-trideoxyTTX	1.4	46	34	3.1	770	44
11-norTTX-6(*S*)-ol	0.49	17	12	8.4	2100	120
11-oxoTTX	0.0072	0.24	0.18	0.026	6.6	0.37

### 2.4. Quantitation of 5,11-DideoxyTTX in the Ovary and Liver of Three Female Specimens of the Pufferfish, *Takifugu pardalis*

The contents of TTX analogs including 5,11-dideoxyTTX in the ovary and liver of three matured female specimens of another Japanese pufferfish, *T. pardalis*, were quantified by high resolution LC-MS (Table 2). 5,11-DideoxyTTX was detected in all samples of ovary and liver, although the amounts of this analog were less than those of 6,11-dideoxyTTX. It is notable that the contents of 5,6,11-trideoxyTTX in the ovary was significantly higher than those in the liver, consistently with our previous report [29].

**Table 2:** The contents of TTXs in the ovary and liver of three female *Takifugu pardalis*.

	Contents (µg/g)	
	ovary ( *n* = 3)	liver ( *n* = 3)
TTX	5.5–27	12–35
4- *epi*TTX	0.34–3.4	1.8–3.0
4,9-anhydroTTX	3.1–12	9.7–16
5-deoxyTTX	0.66–1.0	<0.056–1.4
11-deoxyTTX	0.32–3.4	0.15–0.84
6,11-dideoxyTTX	0.95–6.8	1.0–11
5,11-dideoxyTTX	0.25–0.64	0.26–0.35
5,6,11-trideoxyTTX	6.6–35	0.59–0.87
11-norTTX-6( *S*)-ol	<0.074–5.2	<0.056–0.30
11-oxoTTX	<0.074	<0.056

### 2.5. Toxicity of 5,11-DideoxyTTX to Mice

The toxicity of synthesized (−)-5,11-dideoxyTTX [49] to mice (ddY, male) was examined by intraperitoneal injection. The mice (body weight 18 g) injected with (−)-5,11-dideoxyTTX (10 µg) did not show any symptoms, suggesting that the toxicity of this compound is more than 550 µg/kg.

## 3. Discussion

**Figure 7:**
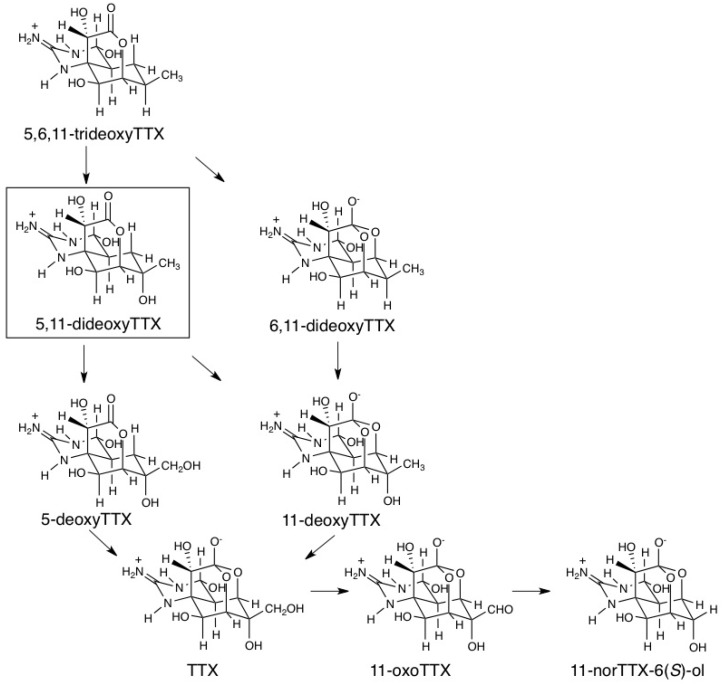
Predicted pathways in the late stages of biosynthesis and metabolism of TTXs.

In the present study, 5,11-dideoxyTTX was identified in two marine animals for the first time by comparison of chromatographic and mass spectrometric properties with those of totally synthesized (−)-5,11-dideoxyTTX. The biosynthetic pathway of TTX still remains unknown. So far, it is difficult to study biosynthesis of TTX using bacteria claimed to produce TTX, because no bacteria have been reported to have the ability to incorporate basic compounds into TTX. Therefore, we have attempted to obtain some information on biosynthesis from the structures of natural analogs of TTX. Since pufferfish and flatworm also contained a series of previously reported deoxy analogs, 5,6,11-trideoxyTTX, 6,11-dideoxyTTX, 5-deoxyTTX, and 11-deoxyTTX, 5,11-dideoxyTTX can be thought as an intermediate of TTX in the oxidation process from 5,6,11-trideoxyTTX to TTX. In this oxidation process, two routes are assumed as shown in Figure 7. One is that 5,6,11-trideoxyTTX is first oxidized to 5,11-dideoxyTTX, which would be oxidized to both of 5-deoxyTTX and 11-deoxyTTX. These monodeoxyTTXs are predicted to be the exact precursors of TTX. The other route is that 5,6,11-trideoxyTTX is first converted to 6,11-dideoxyTTX to be oxidized to 11-deoxyTTX (or 6-deoxyTTX that has not been reported), and then to TTX. We also presume that 11-oxoTTX and 11-norTTX-6(*S*)-ol are oxidized metabolites of TTX. 11-NorTTX-6(*S*)-ol might be a decarboxylation product of 11-carboxylic acid TTX (synthetic analog [50]), possibly derived from 11-oxoTTX, although 11-carboxylic acid TTX has not yet been found in a natural source. In the flatworm, 11-norTTX-6(*S*)-ol is present at high levels, more than TTX in many cases as reported previously. We believe that all these oxidation reactions are proceeded in TTX-producing microorganisms, because we have never detected conversion among TTX analogs in animals by our preliminary attempt so far (data has not been reported).

In the present study, the major fragment ions of 5,11-dideoxyTTX along with those of TTX, 6,11-dideoxyTTX, and 5,6,11-trideoxyTTX were characterized using high resolution ESI-LC-MS/MS. Interestingly, the major fragment ions of TTX and 5,6,11-trideoxyTTX detected at *m/z* 162 as nominal mass by triple quadrupole MS spectrometers were distinguished with each other by high resolution measurement. Since fragment ion (d) in Figure 4 was produced from both TTX and 5,11-dideoxyTTX, and fragment ion (k) was from 5,6,11-trideoxyTTX, 6-OH is suggested to be necessary to produce fragment (d). The fragment ions (g, h, j, and m) were suggested to be the common fragment ions produced by the loss of C10 from all the TTX analogs examined in this study. This data will be useful for further identification of these compounds using high resolution ESI-LC-MS/MS.

For the biological activity, synthesized (−)-5,11-dideoxyTTX was confirmed to be almost nontoxic to mice (minimum lethal dose >550 µg/kg). We previously reported minimum lethal dose of 5,6,11-trideoxyTTX and LD_50_ of 6,11-dideoxyTTX to mice (i.p.) as 750 µg/kg and 420 µg/kg, respectively. Although 6,11-dideoxyTTX still showed 1/42 of the toxicity of TTX (10 µg/kg), the mice injected with (−)-5,11-dideoxyTTX did not show any symptom at 550 µg/kg. The lower toxicity of 5,11-dideoxyTTX than that of 6,11-dideoxyTTX supports the importance of C10-OH and C11-OH in TTX for its voltage-gated Na^+^ channel blocking activity, as previously suggested [50]. 

## 4. Experimental Section

### 4.1. Authentic (−)-5,11-DideoxyTTX, TTX, 6,11-DideoxyTTX and 5,6,11-TrideoxyTTX

Authentic (−)-5,11-dideoxyTTX was totally synthesized [49]. 6,11-DideoxyTTX was purified from the ovary of the pufferfish, *Takifugu pardalis* [31], and TTX and 5,6,11-trideoxyTTX were purified from the pufferfish, *T. poecilonotus* [32].

### 4.2. Preparation of Sample Solutions of Pufferfish and Flatworm for LC-MS Analysis

The sample solution of the ovary of *Takifugu poecilonotus* was prepared from the pooled ovary of the matured female pufferfish captured in Yamaguchi prefecture, Japan [32]. Three ovary and liver samples were individually prepared from three highly matured female specimens of *T. pardalis* purchased from the market in Miyagi prefecture, Japan. The toxins were extracted from homogenized tissues with two volumes of 0.2 M acetic acid (v/v) by heating for 10 min in boiling water. The extract was centrifuged for 15 min at 15,000 rpm at 4 °C, then the supernatant was diluted with five volume of water, and defatted with hexane. After removal of hexane by N_2_ stream, the defatted solution was passed through the reversed phase resin, Cosmosil 75C18-OPN (Nacalai tesque, Kyoto, Japan), packed in a glass pipette and equilibrated with water after washing with MeOH. Then, the passed solution adjusted to pH 6.0 by 1 M NaOH, was loaded on an activated charcoal column (two volumes of the original sample) equilibrated with water. After the column was washed with water (three volumes), TTXs were eluted with acetic acid/EtOH/water (2:50:49, v/v, six volumes). The solvent was removed using a rotary evaporator, the residue was dissolved in 0.05 M acetic acid. An aliquot of this solution was subjected to LC/MS. The pharynx of flatworms (planocerid sp. 1) collected in Guam were lyophilized, and extracted with 0.05 M acetic acid (2 mL/g of flatworm) [10]. Then the extract was treated with charcoal as described above.

### 4.3. High Resolution ESI-LC-MS and LC-MS/MS

The liquid chromatography system used for analysis was a Shimadzu Nexera UHPLC System (Shimadzu, Kyoto, Japan). The autosampler (SIL-30AC, Shimadzu) was kept at 5 °C. Liquid chromatography was performed on a 2.0 i.d. × 150 mm (5 µm) TSK-GEL Amide-80 column (Tosoh, Tokyo, Japan) using 16 mM ammonium formate in water/acetonitrile/formic acid (30:70:0.002, v/v) as a mobile phase at the flow rate of 0.2 mL/min at 25 °C [44]. The liquid chromatography system was connected to a Q-TOF MS spectrometer, MicrOTOFQII (Bruker Daltonics, Bremen, Germany), equipped with an ESI source. The conditions of the MS spectrometer were as following: positive ionization mode, dry gas: nitrogen 7 L/min, dry temperature: 180 °C, nebulizer: 1.6 Bar, capillary: −4500 V. MS/MS was performed in AutoMS/MS mode setting [M + H]^+^ as the precursor ions. The precursor ions and sweeping collision energy were 272.1240 ± 0.1, 33.570–50.358 eV for 5,6,11-trideoxyTTX, 288.1190 ± 0.1, 34.6831–52.046 eV for 5,11-dideoxyTTX and 6,11-dideoxyTTX, and 320.1088 ± 0.1, 36.949–55.423 eV for TTX. 

## 5. Conclusions

By comparison with totally synthesized (−)-5,11-dideoxyTTX, 5,11-dideoxyTTX was detected and exactly identified in pufferfish and flatworm using high resolution ESI-LC-MS/MS. This compound was predicted and now it is supported to be a biosynthetic intermediate of TTX in stepwise oxidation process from 5,6,11-trideoxyTTX to TTX. Furthermore, high resolution masses of the major fragment ions of TTX and its analogs were determined and their corresponding structures were assigned, respectively. These data will be useful for identification of TTXs using high resolution LC-MS/MS.
